# Therapeutic synergies that overcome carboplatin resistance in triple-negative breast cancer

**DOI:** 10.1186/s13046-025-03636-9

**Published:** 2026-02-03

**Authors:** Julia E. Altman, Aaron Valentine, Nina Dashti-Gibson, Emily K. Zboril, David C. Boyd, Rachel K. Myrick, Amy L. Olex, Mikhail G. Dozmorov, J. Chuck Harrell

**Affiliations:** 1 Department of Human and Molecular Genetics, Virginia Commonwealth University, Richmond, USA; 2 Gregory W. Fulton ALS Center Barrow Neurological Institute, School of Life Sciences, Arizona State University, Phoenix, USA; 3 Department of Pathology, Virginia Commonwealth University, Richmond, USA; 4 Department of Biochemistry, Virginia Commonwealth University, Richmond, USA; 5 C. Kenneth and Diane Wright Center for Clinical and Translational Research, Virginia Commonwealth University, Richmond, USA; 6 Department of Biostatistics, Virginia Commonwealth University, Richmond, USA; 7 Massey Comprehensive Cancer Center, Richmond, USA

**Keywords:** Carboplatin, Chemotherapy-resistance, DNA repair, RNA-sequencing, Patient-derived xenograft, Sacituzumab govitecan

## Abstract

**Background:**

Triple-negative breast cancer (TNBC) is an aggressive subtype lacking targeted therapeutic options, where platinum-based chemotherapy such as carboplatin serves as a cornerstone of treatment. Despite initial responses, the rapid emergence of acquired resistance remains a major clinical barrier. Understanding the molecular adaptations that drive platinum resistance is essential to develop strategies to restore sensitivity and identify alternative vulnerabilities.

**Methods:**

We generated four isogenic patient-derived xenograft (PDX) pairs (WHIM30, BCM‑2147, BCM‑3887, BCM‑7482) through serial carboplatin exposure to model acquired resistance in TNBC. Bulk RNA sequencing, immunohistochemistry, and histopathological analyses were performed to define transcriptomic and phenotypic changes associated with resistance. Synergistic therapeutic combinations were identified using high-throughput drug screening in carboplatin-resistant (CR) PDX-derived models, followed by in vivo validation in NSG mice. Tumor growth and survival were assessed using mixed-effects modeling, two-way ANOVA, and Welch’s student t-test.

**Results:**

The resulting isogenic PDX pairs captured both convergent and model-specific adaptations to carboplatin. CR tumors demonstrated heterogeneous activation of DNA damage repair pathways, including restoration of BRCA1-dependent homologous recombination (BCM‑2147, WHIM30) and compensatory upregulation of mismatch repair (BCM‑3887). In the BRCA1-mutant BCM‑7482 model, resistance correlated with HORMAD1 upregulation, suggesting an alternative HRD-associated mechanism. Morphologically, BCM‑7482CR tumors exhibited a significant increase in nuclear size compared to their sensitive counterpart (*p* < 0.0001).

Drug screening identified mTOR pathway inhibition as a recurrent vulnerability across CR models. Sacituzumab govitecan (SG) combined with Everolimus produced robust synergy in vitro and superior tumor control in vivo compared to single agents in both WHIM30CR and BCM‑2147CR. A second combination, Everolimus + Selinexor (KPT‑330), also reduced tumor burden, achieving statistical significance in an expanded WHIM30CR cohort and suppressing metastatic progression in the intrinsically resistant WHIM2 model.

**Conclusions:**

Isogenic PDX models of TNBC provide a powerful platform to define molecular mechanisms of acquired carboplatin resistance and uncover actionable therapeutic strategies. Our findings reveal multiple adaptive routes to platinum resistance, including restoration of homologous recombination and activation of alternative DNA repair programs. Synergistic interactions between SG and mTOR inhibition offer a promising avenue for overcoming resistance, supporting further clinical investigation of these combinations in TNBC.

**Supplementary Information:**

The online version contains supplementary material available at 10.1186/s13046-025-03636-9.

## Background

Breast cancer is the most frequently diagnosed cancer and the second leading cause of cancer-related death in women in the United States [1–3] These malignancies have rich molecular heterogeneity [4–6] and diverse subtypes with distinct pathological responses [4, 7]. Among breast cancer diagnoses, the Triple Negative Breast Cancer (TNBC) subtype, which lacks expression of human epidermal growth factor receptor 2 (HER2), progesterone receptor (PR), or estrogen receptor (ER), are associated with poor patient outcomes [8–10]. TNBC accounts for a significant proportion of breast cancer cases, disproportionately impacting younger women and those with African and Hispanic ancestry [10, 11]. Clinically, TNBCs are considered biologically aggressive and are characterized by relatively short disease-free survival compared to other breast cancer subtypes [12].

The lack of targetable receptors in TNBC limits treatment options, and cytotoxic chemotherapy remains the mainstay of care [13–15]. Carboplatin, a platinum-based chemotherapy agent, has emerged as a frontline treatment option for TNBC due to its ability to induce DNA damage and apoptosis in cancer cells [16, 17]. However, despite its initial effectiveness, the development of acquired resistance to carboplatin remains a significant clinical challenge [18–21]. Resistance mechanisms in TNBC are complex and multifaceted, often involving alterations in a number of key regulatory pathways, making overcoming acquired resistance a difficult task [20, 22]. Further complicating this, TNBCs display rich histological and molecular variations [23, 24]. Recent transcriptomic and proteomic studies highlight both intra- and inter-tumoral diversity within TNBC, underscoring the need to better define its molecular landscape to inform therapeutic development [24–26].

Recent advances in this field underscore the importance of understanding TNBC’s molecular diversity through transcriptomic and proteomic analyses [27, 28]. RNA sequencing and other transcriptomic investigations can provide informative insights into alterations in gene expression patterns and various molecular subtypes found within TNBC models [27, 29]. Meanwhile, proteomic analysis, which makes use of methods such as mass spectrometry, reveals information at the protein level, facilitating the discovery of functional networks and post-transcriptional alterations [30, 31]. Taken together, these omics data can aid in evaluating the information transfer between omics levels and help close the gap between genotype and phenotype [32]. These multi-omic approaches provide a holistic view of TNBC heterogeneity, aiding in the identification of novel therapeutic targets as well as alterations following treatment or acquired resistance.

Although many TNBC patients initially respond to platinum-based chemotherapy, resistance commonly develops [33]. And those patients with residual disease after treatment have poorer prognosis and low survival rates. Elucidating the molecular basis of acquired resistance is essential for identifying strategies to improve patient outcomes by preventing or improving acquired resistance. Prior studies have demonstrated that transcriptional reprogramming accompanies resistance development, making gene expression profiling a powerful tool for uncovering actionable pathways [34, 35].

Patient-derived xenograft (PDX) models serve as a clinically relevant platform to interrogate these mechanisms, as they recapitulate TNBC’s heterogeneity more faithfully than traditional cell lines [36]. In this study, we employ isogenic PDX models of carboplatin resistance generated through successive drug exposure to investigate transcriptional changes at single-cell resolution. By defining the pathways and molecular programs underlying acquired platinum resistance, we aim to identify novel therapeutic targets and inform the development of more effective treatment strategies for TNBC. Given the lack of durable responses to single-agent therapy in this setting, we also sought to evaluate whether other standard-of-care treatments, such as sacituzumab govitecan (SG), retain efficacy in carboplatin-resistant tumors and whether their activity can be enhanced through rational combination with synergistic agents. Integrating these studies with our transcriptional profiling offers the opportunity to identify both mechanisms of cross-resistance and potential vulnerabilities that can be exploited to overcome therapeutic failure in TNBC.

## Materials and methods

### TNBC cell lines & culture conditions

The human TNBC cell lines HCC1143 and MDA-MB-468 were obtained from the American Type Culture Collection (ATCC, Manassas, VA, USA). Cells were routinely cultured in RPMI-1640 medium (Gibco, Thermo Fisher Scientific) supplemented with 10% fetal bovine serum and 1% penicillin-streptomycin (100 U/mL penicillin, 100 µg/mL streptomycin). Cells were maintained at 37 °C in a humidified incubator with 5% CO₂ and routinely monitored for contamination as previously described [10].

### PDX culture

The BCM-2147, BCM-3887, and BCM-7482 PDX models were obtained from Baylor College of Medicine, and WHIM2 and WHIM30 PDX models were sourced from Washington University in St. Louis. All animal experiments were performed under protocols approved by the Institutional Animal Care and Use Committee (IACUC) at Virginia Commonwealth University (Protocol# AD10001247) in accordance with institutional guidelines. Non-obese diabetic severe combined immunodeficient gamma (NSG) mice, bred in-house, were used for all in vivo studies. Tumor cells were suspended in Matrigel (Corning) and injected into the fourth mammary fat pad. Once tumors reached approximately 10 × 10 mm, they were harvested and digested in a solution of DMEM/F12 supplemented with 5% fetal bovine serum (FBS), 300 µL collagenase (Sigma), and 100 µL hyaluronidase (Sigma) [11]. Following digestion, tumors were trypsinized and single-cell suspensions were prepared.

### Generation of carboplatin-resistant isogenic PDX models

Carboplatin-resistant (CR) TNBC PDX models were generated from four parental lines: WHIM30, BCM-2147, BCM-3887, and BCM-7482. For each, matched carboplatin-sensitive (CS) founder tumors were maintained to create isogenic CR/CS pairs. Resistance was induced through serial in vivo passaging under carboplatin treatment (40 mg/kg). Standard dosing consisted of three treatments per passage administered at 3–5 day intervals; however, in some early passages, only one to two doses were given to ensure sufficient tissue for continued passaging. Tumors were designated CR when no significant reduction in tumor volume was observed following treatment as measured with digital calipers. Time to acquire resistance varied between models from 2 to 5 sequential passages. Continued selective pressure by carboplatin dosing was continued for passages after resistance was acquired. Endpoint tumor burden was defined as a maximum dimension exceeding 10 mm in any direction, consistent across all models.

### Single-cell RNA-seq library preparation, quality control and preprocessing

Prior to library preparation, PDX cells were digested into single cell suspensions as previously described [5, 36]. For single-cell collection, cells were dissociated with TrypLE and subsequently resuspended in 0.04% BSA. Single-cell RNA-seq libraries were prepared using the 10x Genomics Chromium Single Cell 3′ v3.1 kit following the manufacturer’s protocol. Target capture was ~ 8,000–10,000 cells per sample. Libraries were quality-checked on an Agilent Bioanalyzer and sequenced at the VCU Genomics Core and samples were sequenced to a minimum read depth of 20,000 reads/cell. Raw sequencing reads were first evaluated for quality using FastQC v0.11.9 and MultiQC v1.11 [37, 38]. Alignment was performed using the 10X Genomics CellRanger v9.0.1 “count” algorithm. For PDX samples, initial alignment was carried out against the 10X Genomics GRCh38_GRCm39-2024-A multi-species genome using the --expect-cells parameter to enable separation of human and mouse cells. Species classification was determined using the CellRanger-generated “gem_classification.csv” file [39]. Barcodes corresponding to human cells were extracted and re-aligned to the 10X GRCh38-2024-A human genome using the --force-cells parameter to retain all identified human cells. Cell-level quality control was conducted using a multi-step adaptive filtering strategy for each sample in R v4.4.1 using Seurat v5 [40]. Briefly, low-quality cells were excluded based on unique molecular identifier (UMI) counts, number of detected genes, and mitochondrial gene expression. Initially, a coarse filter was applied to remove cellular debris and empty droplets by excluding barcodes with fewer than 500 UMIs or 200 detected genes. Subsequently, sample-specific upper thresholds, defined as three median absolute deviations (MADs) above the median for both UMI and gene counts, were used to remove potential doublets calculated on the pre-filtered cell population. The mitochondrial content threshold, set to three MADs above the median and constrained to between 10% and 25%, was calculated using all cells with ≤ 50% mitochondrial content to prevent skew from dying cells. The final set of high-quality cells consisted of those passing all UMI, gene count, and mitochondrial percentage filters (Table 1). Filtered samples were independently normalized using Seurat’s log2 normalization before being scaled with UMI regressed out, and then merged using Seurat’s merge() function. Dimensionality reduction (UMAP and t-SNE), graph-based clustering, and cell cycle analyses were performed as previously described [36]. Loupe-compatible output files were generated using the 10X Genomics LoupeR package (https://github.com/10xGenomics/loupeR).

**Table 1:** Post-filter sample quality information

Sample ID	Number of Cells	Average UMI Count	Average Genes Detected
BCM-2147CR_109178	2710	22786.67	3979.34
BCM-2147_109176	4887	16012.18	3225.63
BCM-3887CR_110347_R2	5812	14679.29	3962.13
BCM-3887_110318_R2	5585	15052.71	3649.04
BCM-7482CR_109078	5311	11113.23	3180.72
BCM-7482_107157	2606	7550.33	2224.29
WHIM30CR_108903	5587	7478.89	2365.11
WHIM30_108896	4967	8149.56	2651.32

### Bulk RNA isolation, quality control and preprocessing

Bulk RNA was extracted from PDX tumor tissue using the RNeasy Mini Kit (Qiagen, Cat. No. 74104) following the manufacturer’s protocol with mechanical disruption and column-based purification. Briefly, approximately 700 µL of RLT buffer was added to freshly dissected tumor fragments, which were mechanically dissociated using autoclaved scissors and homogenized through QIAshredder spin columns. All RNA samples were quantified and assessed for integrity prior to downstream library preparation and sequencing. Library preparation and sequencing were performed by Novogene (Novogene Co., Ltd.). Raw RNA-Seq fastq files were processed by the VCU Massey Comprehensive Cancer Center Bioinformatics Shared Resource (BISR) using an in-house pipeline described previously [5, 8, 41]. Briefly, sequencing quality was assessed using FastQC v0.11.9 [38]. CutAdapt v4.1 [42] removed adapters and low-quality base pairs prior to alignment using STAR v2.7.11a to a merged human (GRCh38)/mouse (GRCm38) genome (for details see Alzubi et al.) using the command line options: “--outSAMtype BAM Unsorted --outSAMorder Paired --outReadsUnmapped Fastx --quantMode TranscriptomeSAM --outFilterMultimapNmax 1. Read counts and log2 TPM values were obtained with Salmon v0.8.2 “quant” algorithm using the “IU” library type [43]. PAM50 subtyping was carried out using the genefu v2.11.2 R package [44].

### Single-cell RNA differential gene expression analysis

Differentially expressed gene (DEG) analysis was performed using the 10X Genomics Loupe Browser (v8.0.0). The “Run Differential Expression” tool was applied with the “Between selected cluster(s) themselves” setting to compare CR and CS tumors. For BCM‑3887, WHIM30, and BCM‑2147 models, time-matched CR and CS tumors were collected and processed together, with library preparation and sequencing performed within the same batch to minimize technical variation. For BCM‑7482, single-cell libraries from CR tumors were generated and sequenced separately from the previously collected CS pair; normalization steps were applied to mitigate batch effects prior to comparison. Results were exported as .csv files containing adjusted p-values, log2 fold changes, and median expression values for all genes excluding those annotated as “low average count” (< 1 count per cell across the dataset).

### Bulk RNA variant analyses

#### Variant calling

Aligned BAM files were processed on the Virginia Commonwealth University high-performance computing cluster. The human reference genome GRCh38 (primary assembly) was used, with “chr” prefixes removed for compatibility with downstream tools. BAM files were sorted and indexed using samtools v1.21 prior to variant calling. Variants (SNPs and indels) were called using bcftools mpileup and bcftools call with multiallelic calling enabled [45]. Raw VCF files were compressed and indexed with bcftools index. To reduce false positives, variants were filtered with thresholds QUAL > 20, DP > 10, and MQ > 40, and filtered VCFs were indexed for downstream analyses. RNA-seq variant calls may include RNA-editing events, typically A to G or T to C substitutions. These artifacts are minimized in our pipeline by: read-quality and mapping-quality requirements in bcftools mpileup/call, high-confidence filtering (QUAL ≥ 20, MQ ≥ 40) which removes the majority of low-allele-fraction RNA editing signals, depth requirement (DP ≥ 10) to exclude low-level editing events, and sensitive/resistant comparisons, which treat variants conservatively and do not interpret low-level, non-reproducible mismatches as biological differences.

Commands used were:

$$\begin{aligned} &bcftools\:mpileup-Ou-f\:GRCh38.nochr.fa\:sample.bam\\&\mid{bcftools\:call-m-Oz-o\:sample.raw.vcf.gz} \end{aligned}$$

$$\begin{aligned} &bcftools filter-e\,^{\prime}QUAL<20||DP<10||MQ<40^{\prime} \\&\backslash-Oz-o\,sample.filtered.vcf.gz\:sample.raw.vcf.gz \end{aligned}$$

#### Variant annotation and comparison

Filtered VCFs were imported into R using the VariantAnnotation package v.1.54.1 [46]. Gene coordinates were obtained from Gencode v43 annotations via a TxDb object, and gene symbols were mapped to Ensembl IDs using org.Hs.eg.db. Variants were annotated for clinical significance using a ClinVar VCF (hg38, bgzipped and indexed). For each gene of interest (BRCA1, BRCA2, TP53), variants within the gene region were extracted from each sample VCF. Variants were classified as shared or unique between paired samples, and genotypes were compared side-by-side in a wide-format table. ClinVar pathogenicity annotations were added for each variant to identify previously reported disease-associated alleles. The final output was saved as CSV tables for downstream analyses.

### Differential expression analysis of bulk RNA-seq data

Raw count data from bulk RNA-seq were imported into R (v4.3.2) for preprocessing and differential expression analysis. Count matrices were read in as .txt files and curated to standardize column names, parse sample identifiers, and remove non-coding transcripts. Genes with zero counts across all samples were excluded from downstream analyses. Differential expression analysis was performed using the edgeR v4.0.16 package in R [47]. Count data were filtered to retain only genes with counts per million > 1 in at least two samples. Library sizes were recalculated, and normalization was performed using the trimmed mean of M-values method. Multi-dimensional scaling plots were generated to visualize sample clustering based on biological coefficient of variation. For group comparisons, a design matrix was constructed based on PDX model identity. Common, trended, and tagwise dispersions were estimated using edgeR’s negative binomial model framework. Differential expression was assessed using the exactTest function in edgeR, comparing CR versus carboplatin-sensitive CS tumors within each PDX model. Resulting p-values were adjusted for multiple testing using the Benjamini–Hochberg method to control the false discovery rate (FDR). Genes with FDR < 0.05 were considered differentially expressed. Significant genes were visualized using smear plots. Gene-level results for each PDX pairwise comparison were exported as CSV files.

### Nuclear area comparisons in BCM-7482

#### Nuclear area quantification

Formalin-fixed paraffin-embedded (FFPE) tumor sections from BCM‑7482 CS and CR PDX tumors were imaged using the Agilent Cytation 7 imaging system at 20× magnification. Automated nuclear segmentation was performed using the Gen5 Image + software (Agilent), with a DAPI channel mask applied to define nuclear regions of interest. Identical thresholding parameters and segmentation settings were applied to all images to ensure consistency between CS and CR samples. For each image, the software exported per-object nuclear measurements including area, integrated intensity, and shape descriptors. Data from multiple images across biological replicates were combined for downstream analysis.

#### Data processing and statistical analysis

Raw nuclear object measurements were exported into .xlsx format. Nuclear area was extracted and averaged per image, and distributions of nuclear sizes were compared between CS and CR tumors. Statistical significance was determined using Welch’s t-test to account for unequal variance between groups. All analyses and visualization of bar plots were performed in GraphPad Prism v10.6.0.

### Immunohistochemistry (IHC) staining

FFPE sections were baked at 60 °C for 30 min and sequentially deparaffinized in xylene and graded ethanols prior to rehydration in deionized water. Antigen retrieval was performed in 10 mM Tris-EDTA buffer (pH 9.0) using a pressure cooker, followed by gradual cooling to room temperature. Endogenous peroxidase activity was quenched using a commercial peroxidase block, and sections were incubated with rabbit primary antibodies overnight at 4 °C in a humidified chamber. Following washes in TBST, sections were incubated with HRP-conjugated polymer detection reagent for 20 min, developed using DAB substrate, and counterstained with hematoxylin. Slides were dehydrated through graded ethanols, cleared in xylene, and mounted with Permount mounting medium (Table 2).

**Table 2:** Primary antibodies used for IHC staining

Protein Target	Isotype	Reactivity	kDa	Dilution	Company	Catalog Number
BRCA1	Rabbit IgG	H, Mk	220	1:200	Cell Signaling Technology	#50,799
Cytokeratin Pan Polyclonal	Rabbit IgG	H, M	40–68	1:1000	ThermoFisher Scientific	#PA1-27114
Ki-67	Rabbit IgG	H		1:400	Cell Signaling Technology	#9027
Phospho-Histone H3	Rabbit IgG	H, M, R, Mk, Dm	17	1:200	Cell Signaling Technology	#9701
UCHL1	IgG	H, M, R, Mk		1:400	Cell Signaling Technology	#13,179
RAB31	IgG	H, M, R		1:50	ProteinTech	#16182-1-AP
NID1	IgG	H		1:100	GeneTex	#GTX114587
COL4A2	IgG	H		1:1000	ProteinTech	#55131-1-AP
FBLN1	IgG	H, M, R		1:500	Novus	#NBP1-84726
TYMP	IgG	H		1:500	ProteinTech	#12383-1-AP
ANPEP	IgG	H, M, R		1:500	ProteinTech	#14553-1-AP
EGFR	IgG	H, M, Mk		1:50	Cell Signaling Technology	#4267

### Immunohistochemistry (IHC) image quantification

Quantification of IHC staining was performed using Fiji (ImageJ) across biological replicates, with a minimum of three representative images analyzed per tumor [48]. TIF or PNG images were imported into Fiji and processed using the Color Deconvolution plugin with the “H DAB” vector setting. Following deconvolution, the DAB signal (Color 2) was selected, and thresholding was applied under *Image > Adjust > Threshold*, ensuring the “Dark Background” option was unchecked. Thresholds were set with the minimum value fixed at 0, and the maximum adjusted manually to isolate DAB-positive staining. The mean intensity of the DAB signal was then recorded using the *Analyze > Measure* function.

Subsequently, the hematoxylin signal (Color 1) was selected to quantify nuclei. The image was thresholded using the same parameters (minimum = 0, maximum adjusted), followed by application of *Process > Binary > Fill Holes* and *Process > Binary > Watershed* to improve nuclear segmentation. Particle analysis was performed under *Analyze > Analyze Particles*, with the size parameter set to exclude small artifacts (minimum size: 51 pixels, approximating the average nuclear diameter). The total nuclei count per image was recorded. For each image, the mean DAB intensity was normalized to the number of nuclei by dividing the DAB signal by the nuclei count, generating a per-cell intensity estimate for each replicate. Final ratios were multiplied by 100 for axis readability. Protocol is an adapted version of prior published method [49].

BRCA1 IHC comparison significance was calculated using linear mixed model with Satterthwaite’s method in R using lmerTest package [50]. Of note, IHC images quantified for WHIM30 and BCM-2147 CS/CR models for proteomic validation were done on 4–5 representative images from a single biological sample, and significance was calculated using unpaired student t-test in Prism by GraphPad.

### Drug combination and synergy analysis

High throughput drug screening was performed as previously described using a library of 555 compounds (NCI NExT Oncology Interrogation Tools Library) at a concentration of 1 µM [4, 5, 36, 51] in combination with the IC20 dose of sacituzumab govitecan (Supplemental File 5). Drug combination experiments were conducted to assess potential synergy between SG and secondary agents in CR PDX-derived tumor models. Sacituzumab govitecan was tested at four concentrations (0–40 nM) in combination with eight-point dose ranges (0–100 µM) of Everolimus, Talazoparib, Selinexor (KPT-330), or Ribociclib. Cell viability was measured after 72 h of treatment using the CellTiter-Glo luminescent assay [52]. Dose–response matrices were analyzed using the SynergyFinder platform to calculate synergy scores based on the BLISS reference model [53].

### Protein extraction and quantification

Freshly flash-frozen tumor fragments were lysed in buffer containing 8 M urea, 1% SDS, and 200 mM EPPS (pH 8.0) supplemented with protease and phosphatase inhibitor cocktails. Lysates were sonicated on ice to ensure complete disruption and solubilization of proteins. Protein concentrations were determined using the Bradford method according to the manufacturer’s instructions. For each sample, 200 µg of total protein was aliquoted and submitted for total proteome analysis to the Thermo Fisher Scientific Center for Multiplexed Proteomics at Harvard Medical School (https://tcmp.hms.harvard.edu).

### Proteomic data analysis

Differential proteins were identified using log2 protein abundances and the limma v.3.64.3 R package (Supplemental File 4) [54]. Proteomic pathway enrichment was performed using two complementary approaches: Enrich (over-representation analysis) and GSEA (rank-based enrichment using ordered fold changes). KEGG pathways and MSigDB gene-set collections (H and C1–C7) were used. Results are provided in Supplemental Files 2 & 3 with worksheets labeled by analysis and signature (e.g., Enrich.KEGG, GSEA.C7).

### In vivo drug treatment studies in PDX models

Therapeutic studies were performed using CR PDX models WHIM30CR and BCM-2147CR, as well as the intrinsically carboplatin-resistant WHIM2 model. For in vivo expansion and propagation, tumor cells were prepared and implanted as previously described. Briefly, 5 × 10^5 tumor cells suspended 1:1 in Cultrex basement membrane extract (Bio-Techne) were injected bilaterally into the fourth mammary fat pads of female NSG mice (500,000 cells per injection) as previously described [5]. Tumors were allowed to establish for approximately three weeks (~ 21 days) before treatment initiation. To minimize baseline variability, mice were informally checked to ensure that tumors were of comparable size across groups prior to enrollment.

#### Vehicle formulation

For in vivo studies, drugs were either dissolved in 1% methylcellulose + 0.1% Tween-80 and administered via oral gavage or saline to be administered through intraperitoneal injection as indicated.

#### Group sizes and experimental cohorts

For WHIM30CR, the initial cohort consisted of 3 mice per group; an expanded cohort of 4 mice per group was subsequently performed to evaluate the Everolimus + Selinexor (KPT-330) combination. BCM-2147CR cohorts included 4 mice per group. For WHIM2 mammary gland tumor (MGT) studies, groups included vehicle (*n* = 4), Selinexor (KPT-330) (*n* = 4), Everolimus (*n* = 5), and combination (*n* = 5). For WHIM2 metastasis studies (intracardiac injection), groups consisted of vehicle (*n* = 4), Selinexor (KPT-330) (*n* = 3), Everolimus (*n* = 3), and combination (*n* = 4).

#### Drug administration

Sacituzumab govitecan (SG) was administered intraperitoneally once weekly at 5 mg/kg. Everolimus was given by oral gavage 10 mg/kg (in 100 µL) three times per week. Selinexor (KPT-330) was administered by oral gavage at 5 mg/kg (100 µL) three times per week. For combination groups, agents were given on the same schedules as their respective monotherapy arms. Vehicle groups received the corresponding vehicle formulation(s) on the same schedule. Carboplatin (40 mg/kg, three doses per passage at four-day intervals) was used for the generation of resistant PDX lines, but was not used in subsequent combination efficacy experiments.

#### Tumor monitoring and humane endpoints

Tumor dimensions were measured twice weekly using digital calipers, and tumor volume was calculated as (length × width^2) / 2, where width is the smaller dimension. Mice were euthanized when tumors exceeded 15 mm in any direction, when body weight loss exceeded 20%, or at planned experimental endpoints. In experiments where vehicle-treated tumors reached burden earlier than treated groups, those mice were euthanized and remaining treatment cohorts continued until endpoint criteria were met.

#### WHIM2 metastasis protocol and imaging

To assess metastatic burden, WHIM2 cells (500,000 cells in 100 µL HF buffer) were injected intracardially into five-week-old NSG mice. Successful injections were confirmed immediately using IVIS^®^ Spectrum imaging (PerkinElmer) with Living Image^®^ 4.7.4 software. After 35 days of treatment, mice were injected intraperitoneally with 200 µL luciferin (150 mg/kg), incubated for 5 min, and imaged for whole-body metastasis. Brains and ovaries were subsequently harvested and imaged ex vivo on the IVIS. Metastatic burden was quantified by organ radiance, and radiance values were normalized across groups to compare metastatic load between treatments.

#### Statistical analysis

For the initial WHIM30CR cohort (all mice harvested simultaneously), Welch’s two-sample t-test was used to compare tumor volumes. For the WHIM30CR expansion cohort and BCM-2147CR studies, where vehicles reached endpoint earlier than treatment groups, tumor growth trajectories were analyzed using linear mixed-effects models. The fixed-effects structure was specified as Measurement ~ Days * Condition, with random intercepts for Mouse and for Mouse: Side (nested), and significance of the Days: Condition interaction was used to test treatment effects. The standardized effect size was calculated as the interaction estimate divided by the model residual SD. For WHIM2 MGT and metastasis studies, tumor volume and organ radiance were compared between groups using one-way ANOVA, with *p* < 0.05 considered significant.

## Results

### Generation of isogenic platinum-resistant PDX models

**Fig. 1:**
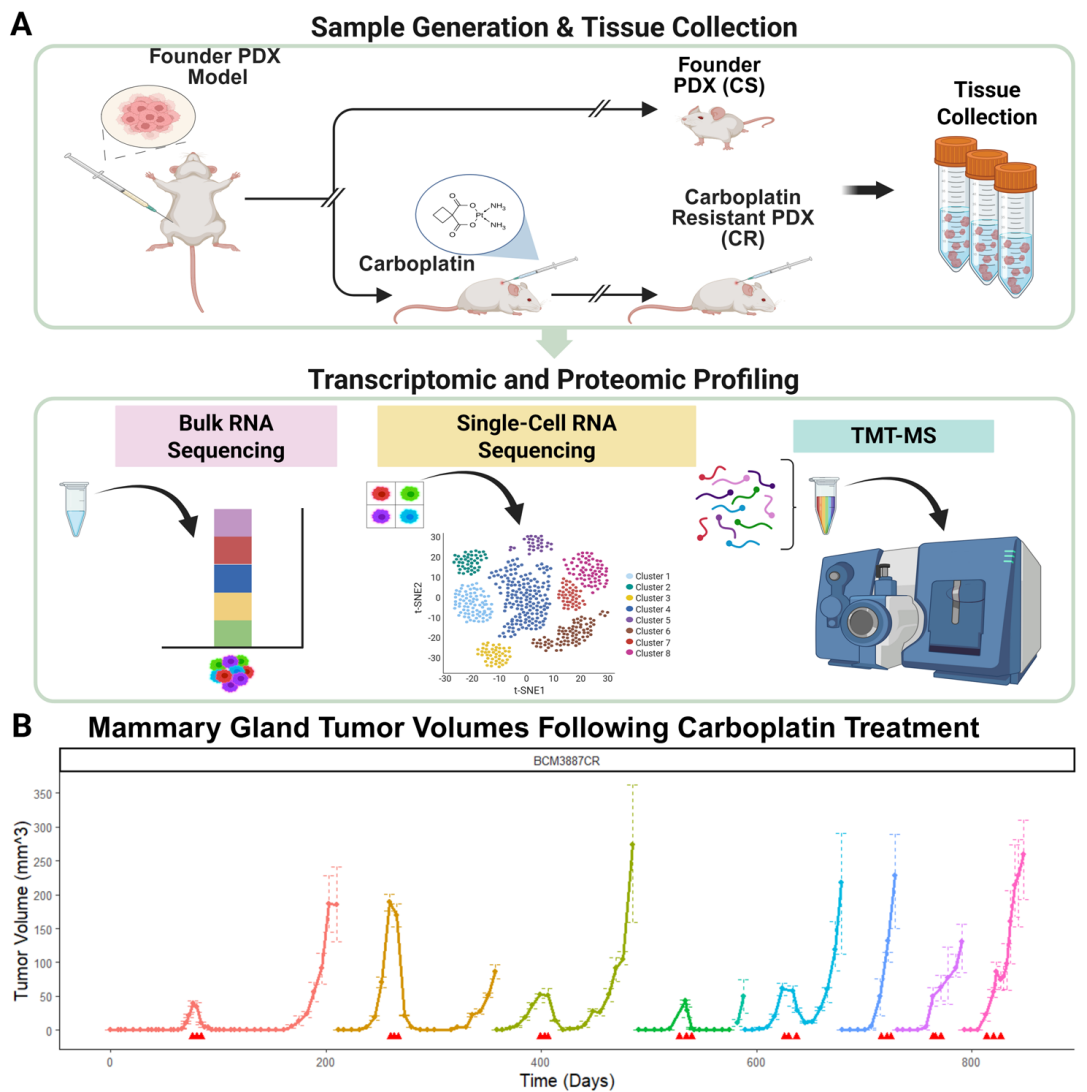
Model generation & development.** A** Outline of tissue generation methods and subsequent profiling techniques. **B** Tumor volume graphs for PDX model (BCM-3887 shown), beginning with cells from the founder PDX and carried through serial passages with applied carboplatin treatments. *Intraperitoneal (IP) injection of 40 mg/kg of carboplatin is indicated by red arrows. Serial passage of cells into new mice is represented by a new graph segment and color*

The novelty of this study lies in the generation of isogenic paired TNBC PDX models of carboplatin resistance from four founder lines (WHIM30, BCM-2147, BCM-3887, BCM-7482). All four lines were initially characterized to be CS, showing notable reduction in primary tumor volume following treatment. Through serial in vivo passaging under carboplatin treatment, CR variants were established (Fig. 1A). Resistance was defined as the absence of a significant reduction in tumor mass after treatment. Across the four PDX lines, resistance was achieved after 2–5 passages (Fig. 1B). This approach yielded four matched CR/CS PDX pairs encompassing diverse TNBC backgrounds.

These paired models provide a controlled system for dissecting resistance mechanisms. Once resistance was confirmed, tumors from both CS and CR lines were collected for downstream omics analyses, including bulk and single-cell RNA sequencing and tandem mass tag mass spectrometry (TMT-MS) (Fig. 1A). These datasets also offer a clinically relevant platform for further testing rational drug combinations with potential synergy alongside standard therapies.

### Histopathological and morphological differences between isogenic CR/CS PDX pairs

**Fig. 2:**
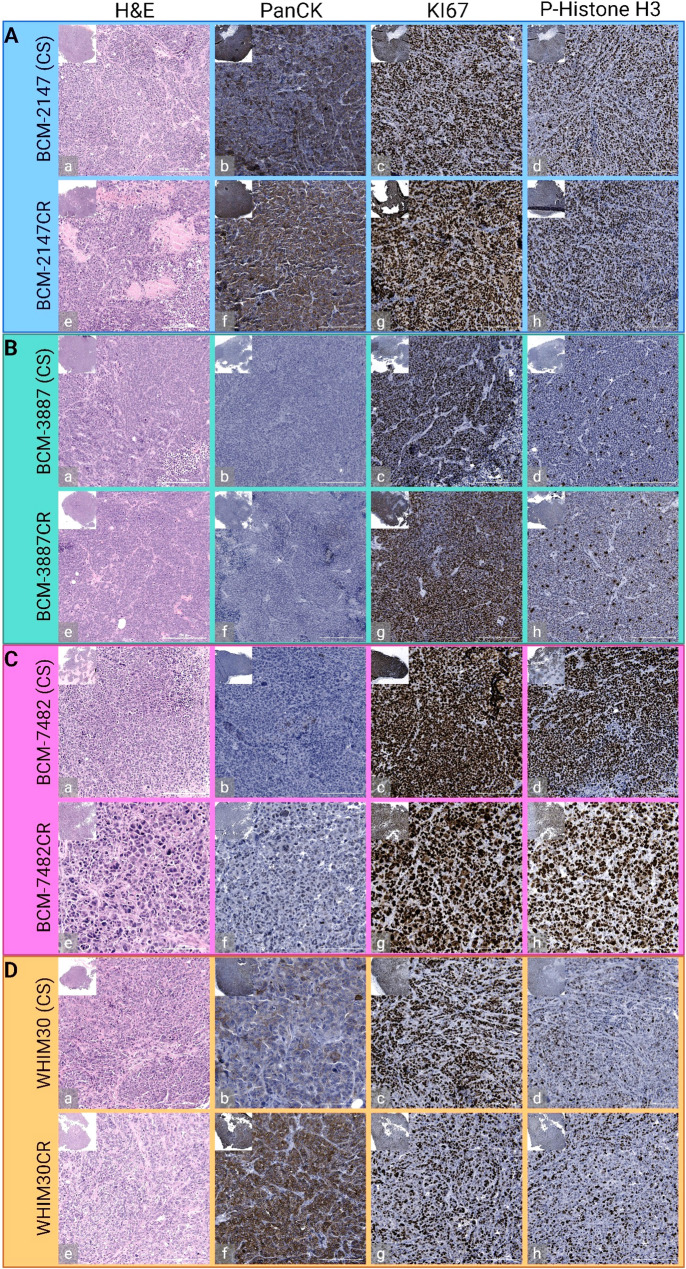
Immunohistochemical staining of representative sections within CR and CS pairs. Histological staining of (**A**) BCM-2147 (**B**) BCM-3887 (**C**) BCM-7482 and (**D**) WHIM30 CS (a-d) and CR (e-h) model pairs with representative images taken at 20x magnification. For each model simple hematoxylin and eosin staining (a, e) was performed alongside immunohistochemical stains for pan-cytokeratin (b, f), Ki67 (c, g), and phospho-histone H3 (d, h)

Following model generation, tumors were resected for CR/CS pairs for histology. Histological and immunohistochemical evaluation revealed both shared and model-specific phenotypic changes between CR and CS tumors. Across all four PDX pairs (BCM‑2147, BCM‑3887, BCM‑7482, and WHIM30), hematoxylin and eosin (H&E) staining demonstrated preserved overall tumor architecture with variable regional necrosis (Fig. 2A-D: a, e). Pan‑cytokeratin (PanCK) staining confirmed maintained epithelial identity in both CR and CS tumors for BCM-2147 (Fig. 2A: b, f) and WHIM30 (Fig. 2D: b, f) models, with no appreciable loss of cytokeratin expression following resistance. BCM-3887 (Fig. 2B: b, f) and BCM-7482 (Fig. 2C: b, f) models maintained low PanCK staining levels prior to and following resistance. Ki67 and phospho-histone H3 (pHH3) staining did not show consistent changes in proliferative indices between CR and CS models (Fig. 2A-D: c, d, g, h).

Upon initial inspection of BCM‑7482 tumor sections under 20× magnification, CR tumors appeared to contain noticeably larger cells/nuclei than CS tumors, prompting quantitative analysis. Since there were clear differences in the size of tumor cells within BCM-7482CR, automated nuclear segmentation of BCM‑7482 tumor sections was performed and a significant increase of nuclear size within CR tumors compared to their CS counterparts was observed (Supplemental Fig. S1; Supplemental File 1). Quantification of segmented nuclear objects demonstrated a significant shift in mean nuclear area in CR tumors, consistent across multiple images and biological replicates (*p* < 0.0001, Unpaired t-test). This nuclear enlargement aligns with the striking morphological differences observed in H&E sections of BCM‑7482CR tumors, supporting the phenotypic distinction between CR and CS pairs in this model. No notable differences in nuclear size or morphology were observed in the three other basal-like TNBC models.

### Single-cell transcriptional divergence accompanies carboplatin resistance in TNBC PDXs

**Fig. 3:**
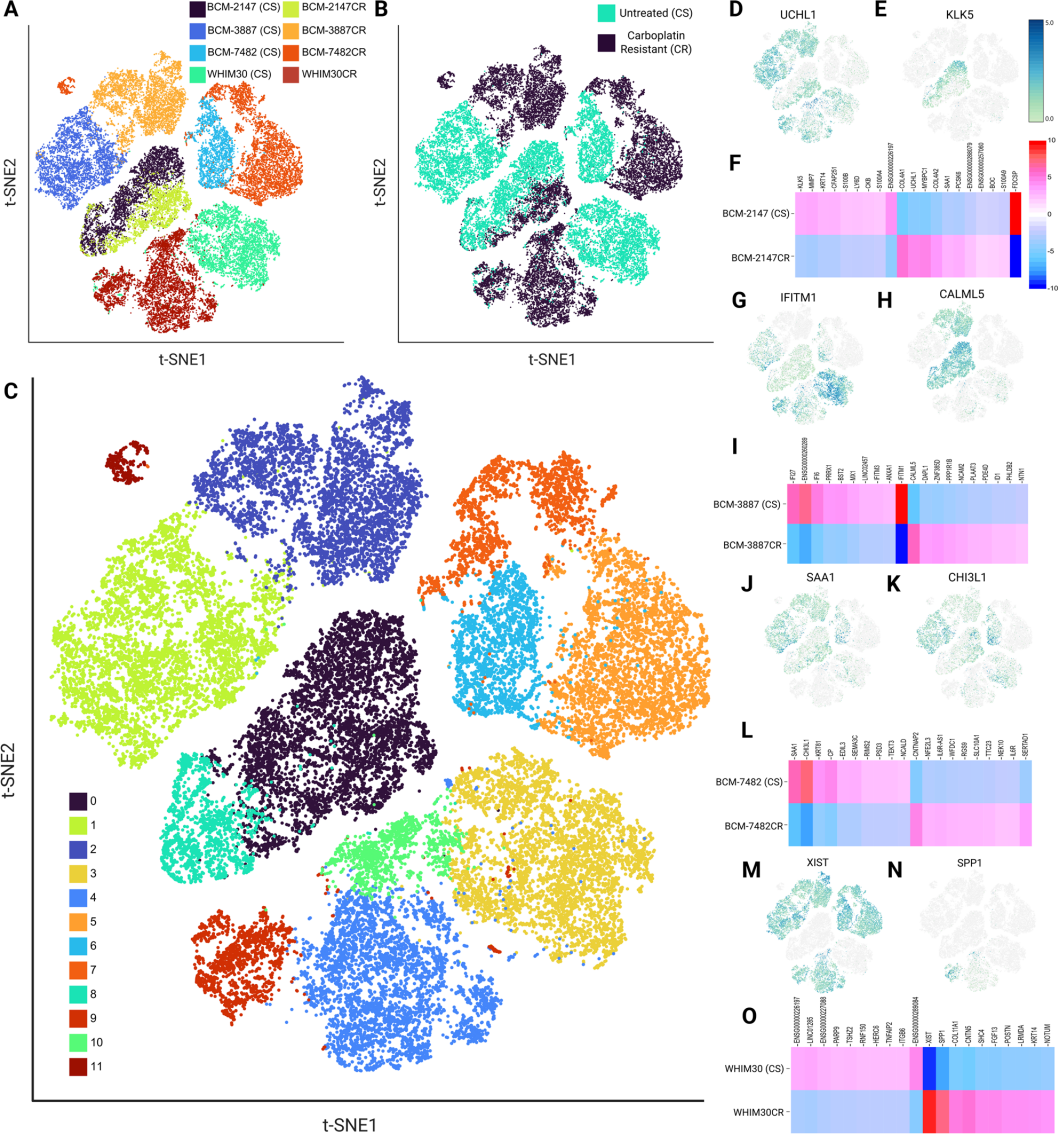
Single cell RNA sequencing analysis of CS/CR pairs. T-SNE embeddings of single-cell RNA-seq data from matched carboplatin-resistant (CR) and carboplatin-sensitive (CS) tumors across four PDX models colored by (**A**) sample, (**B**) treatment, and (**C**) Seurat-derived transcriptional clusters. **F**,** I**,** L**,** O** Heatmap plots of DEGs for each model, illustrating distinct sets of up- and down-regulated transcripts and tSNE plots corresponding to select DEGs (**D**-**E**,** GH**,** J**-**K**,** M-N**). Significance thresholds were defined by FDR < 0.05

To define transcriptional changes associated with CR at the single-cell level, we performed scRNA-seq on matched CR and CS tumors (Fig. 3A). In t-SNE embeddings, CR cells within each model occupied distinct transcriptional space from their paired CS cells, yet CR cells were more similar to their CS pair than to CR cells from other models (Fig. 3A-B). These patterns indicate that transcriptionally distinct profiles emerged during resistance development, while overall, each PDX maintained its unique genomic identity.

To assess cellular heterogeneity within and between models, we performed Seurat clustering and visualized the resulting clusters using tSNE embeddings (Fig. 3C). CS and CR cells projected into multiple discrete transcriptional sub-clusters, indicating substantial intra-tumoral heterogeneity. Each PDX pair shared at least one major clusters, although the relative proportions of cells within these clusters differed between sensitive and resistant tumors. While some clusters within WHIM30 and BCM-7482 segregated almost exclusively CR or CS cells (clusters 3–4 & 5–6), BCM-2147 and BCM-3887 model pairs showed less strict cluster segregation based on resistant status alone. Together, these data indicate that carboplatin resistance can arise through different modes across models, ranging from shifts in the abundance of pre-existing transcriptional states to the apparent emergence of distinct, resistance-associated clusters.

Differentially expressed gene (DEG) analysis was performed within each CS/CR pair to identify model-specific transcriptional changes. Each PDX exhibited a unique set of up- and down-regulated genes, reflecting distinct molecular programs driving resistance. No individual genes were significantly altered across all models after multiple testing correction, highlighting the heterogeneity of CR mechanisms (Fig. 3D-O). Nonetheless, subsets of genes were shared among two or more models, suggesting partial convergence of resistance pathways.

### Homologous repair related pathways in acquired carboplatin resistance

**Fig. 4:**
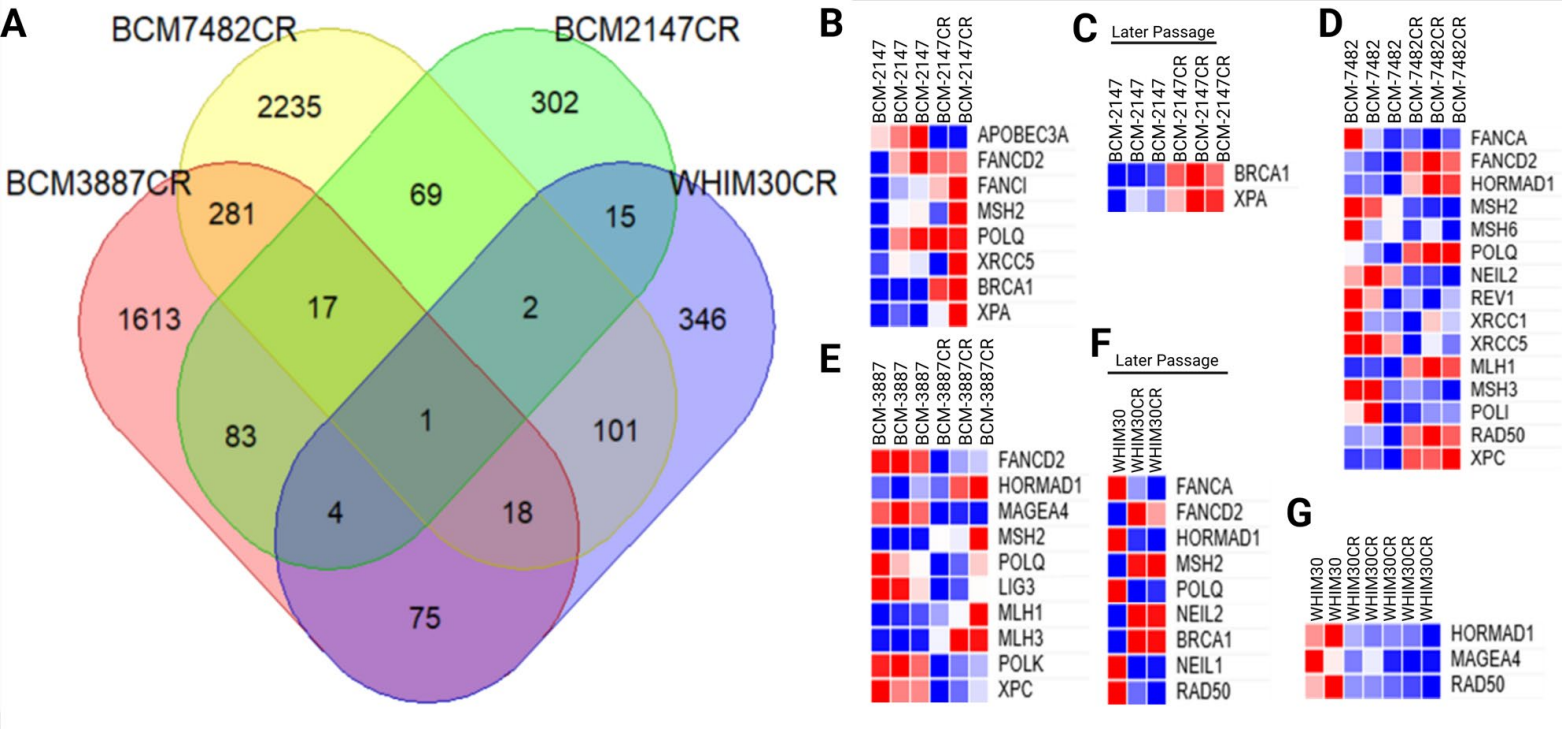
Homologous recombination–related pathways and differential gene expression in carboplatin-resistant TNBC models. **A** Venn diagram showing overlap of differentially expressed genes (DEGs) between carboplatin-resistant (CR) and carboplatin-sensitive (CS) tumors across the four isogenic PDX models (WHIM30, BCM-2147, BCM-3887, BCM-7482). Representative plots illustrating HRD related genes in CR tumors relative to CS counterparts for (**B–C**) BCM-2147, **D** BCM-7482, **E** BCM-3887, and (**F-G**) WHIM30. Data represent RNA-seq analyses, with significance defined by FDR < 0.05. *“Later passage” refers to batch sequenced samples from subsequent serial passaging of the CR model after the initial set of samples*,* having been under selective pressure from carboplatin treatment longer. Each column represents an independent biological replicate*

Bulk RNA sequencing of the paired CS and CR PDX models was performed following scRNAseq. From the bulk RNA profiles, DEG analysis was performed in R for each model set. When lists of differently expressed genes were compared across models, there was some overlap; however, after multiple testing correction there was only one slightly upregulated gene that overlapped in all 4 CR/CS model comparisons (Fig. 4A). The identified gene was SIX3, a member of the sine oculis homeobox transcription factor family that has been associated with cancer signaling [55, 56].

Since prior literature suggests that homologous recombination deficiency (HRD) can be linked to resistance mechanisms for DNA damaging agents and carboplatin is commonly prescribed to patients with BRCA1 mutations, we sought to stratify genomic changes that occurred during CR with particular emphasis on BRCA1- mediated repair and alternative HRD-related pathways [57, 58]. The DEG analyses were contrasted with known HRD genes [59]. Within the BCM‑2147 pair, the CR tumors exhibited a marked increase in BRCA1 expression relative to the CS line (Fig. 4B-C). This transcriptional upregulation suggests restoration or enhancement of BRCA1-dependent homologous recombination as a primary driver of acquired resistance. This same trend was observable under IHC probing for BRCA1 (Supplemental Fig. 2; p-value < 0.1). Consistent with this, variant interrogation of bulk RNA sequences did not detect BRCA1 mutations in this model, supporting a functional repair axis. In WHIM30, we identified heterogeneity between different CR passages (Fig. 4F-G). One set of CR derivatives demonstrated significant BRCA1 upregulation, implicating the same BRCA1-mediated homologous repair pathway in resistance. In contrast, an earlier CR derivative lacked this BRCA1 increase but had consistent downregulation of HORMAD1 and MAGE‑A4. Both transcripts are cancer-testis antigens associated with DNA damage tolerance and have been reported as elevated in HRD-high breast tumors. Their suppression in WHIM30CR suggests a possible shift away from this alternative HRD mechanism, indicating more than one route to resistance within this model. For BCM‑3887, which harbors a pathogenic splice-site BRCA1 mutation resulting in loss of functional protein [60], CR tumors did not show increased BRCA1 expression (Fig. 4E). Instead, we observed significant upregulation of mismatch repair genes MLH1, MLH2, and MLH3 in the resistant tumors. These findings suggest that, in the absence of a functional homologous recombination axis, enhanced mismatch repair may underlie carboplatin resistance in this model.

The BCM‑7482 model, carrying a pathogenic BRCA1 frameshift mutation [61], similarly lacks a functional BRCA1 repair pathway (Fig. 4D). Here, CR tumors showed strong upregulation of HORMAD1 compared to CS tumors, consistent with an adaptive increase in DNA damage tolerance and replication stress response. This claudin-low model displayed a broader DEG profile in HRD-related genes but without a uniform trend across the canonical homologous recombination pathways, highlighting a potentially distinct resistance biology.

A variant analysis was performed on the bulk RNA sequencing data for BRCA1, BRCA2, and TP53. While known mutations in TP53 were observable by this analysis in BCM-7482 and BCM-3887, no novel mutations related to these genes was observed in CR models when compared to their CS counterpart.

### Candidate resistance-associated pathways identified by proteomic profiling

Having identified some potential routes of resistance in these models, we sought to determine if any common targetable pathways existed. We performed Tandem Mass Tagged Mass Spectrometry performed on the BCM-2147 and WHIM30 CS/CR sets. Of note, several proteins were seen to be significantly different between CR and CS pairs in these models (Supplemental File 4). We further validated some of those targets with IHC characterization in relevant tumor sections (Supplemental Fig. 3).

To further identify coordinated biological processes associated with carboplatin resistance, we performed pathway enrichment analysis on the differentially expressed proteins. Both over-representation analysis (Enrich) and rank-based gene set enrichment analysis (GSEA) were applied using KEGG pathways and MSigDB collections (H, C1–C7). Across models, carboplatin-resistant tumors showed differential enrichment of several metabolic and stress-response pathways—including glycolysis/gluconeogenesis, amino sugar and nucleotide sugar metabolism, and glutathione metabolism—as well as pathways related to extracellular matrix organization such as focal adhesion and ECM–receptor interaction, providing pathway-level context for the proteomic differences between CR and CS tumors (Supplemental Files 2 & 3).

We identified several genes which we thought might be promising in our models, including UCHL1, an emerging target in breast cancer [62]. Despite this, preliminary testing of inhibitory agents targeting these proteins in vivo largely failed to produce meaningful effects on reducing overall tumor burden, indicating that while these proteins may mark resistant tumors, more testing is needed to fully determine their utility as direct therapeutic targets in these models.

### Synergistic therapeutic strategies in CR TNBC models

Beyond characterizing pathways associated with carboplatin resistance, these models also serve as a clinically relevant platform for testing rational drug combinations, including the evaluation of synergistic agents alongside current standard of care. For this reason, we pursued identifying agents which would work synergistically with the standard care agent SG, a TROP2-targeted antibody-drug conjugate currently in clinical use [63, 64].

**Fig. 5:**
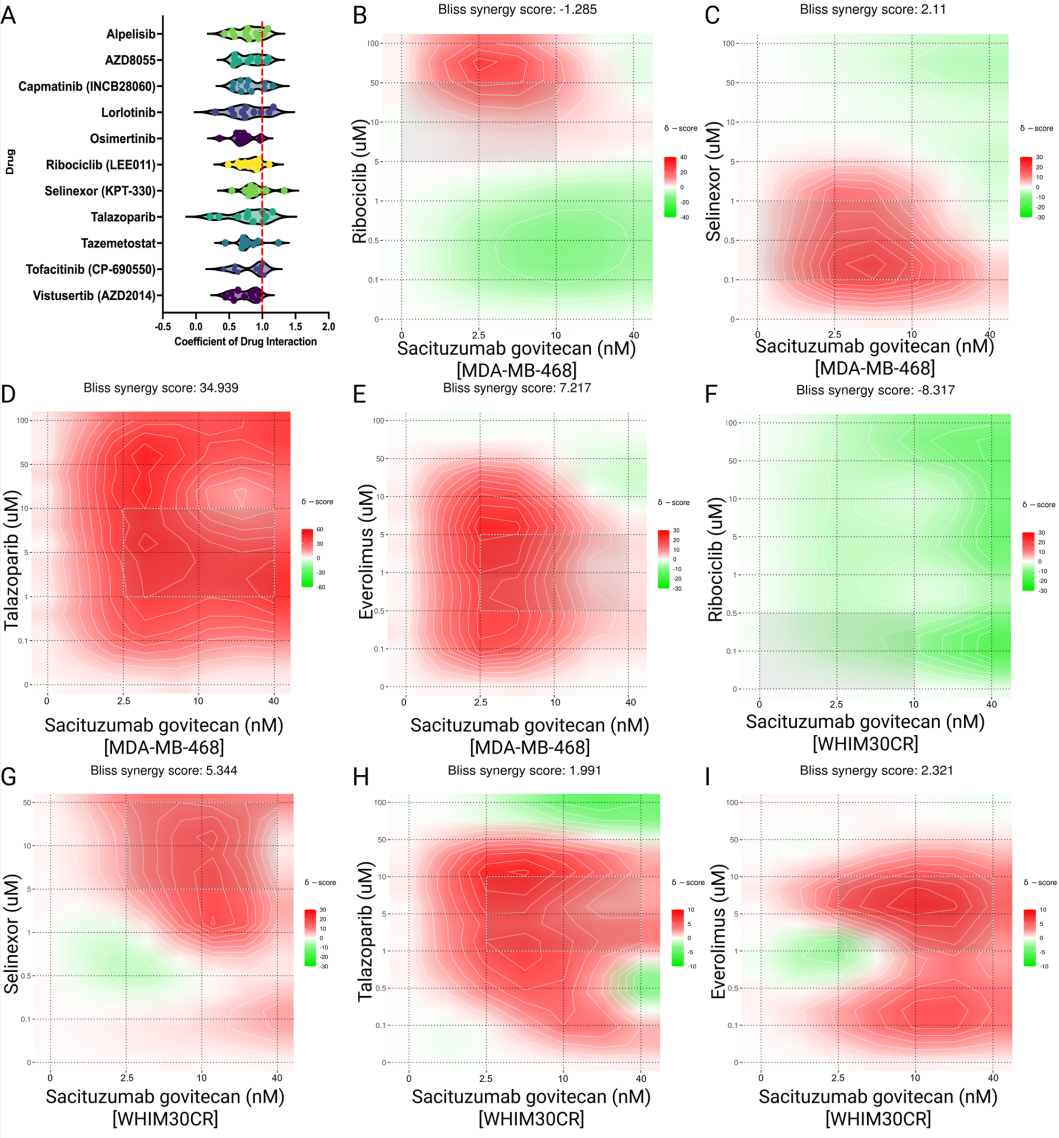
High-throughput drug screening identifies synergistic vulnerabilities in carboplatin-resistant TNBC.** A** Violin plots of calculated coefficient of drug interaction (CDI) for top synergistic drugs across multiple ex vivo PDX and cell line models, highlighting synergistic interactions between SG and mTOR inhibition. **B**–**I** Dose response matrices (BLISS model) from in vitro combination assays in WHIM30CR and MDA-MB-468 cells, showing enhanced growth inhibition with SG plus Everolimus, Selinexor (KPT-330), or other targeted agents. Cell viability was measured after 72 h using CellTiter-Glo. Synergy was reproducible across independent experiments

To identify actionable vulnerabilities in CR TNBC, we performed high-throughput drug screening with an emphasis on synergistic interactions with SG. Across models, several strong synergistic interactions emerged, with several mTOR pathway inhibitiors consistently ranking among the top candidates when quantified by coefficient of drug interaction (CDI; values < 1 indicate synergistic effects) (Fig. 5A & Supplemental File 5; Vistusertib (AZD2014) & AZD8055 shown).

Drug combination assays were carried out in vitro in WHIM30CR model and MDA-468 cell line using a four-point SG dose range (0–40 nM) in combination with eight-point dose ranges (0–100 µM) of Everolimus, Talazoparib, Selinexor (KPT-330), or Ribociclib (Fig. 5B-I). Cell viability was assessed after 72 h using the CellTiter-Glo assay.

Everolimus, an FDA-approved mTORC1 inhibitor with established clinical utility, demonstrated robust and reproducible synergy with SG in multiple CR lines (Fig. 5E, H). Notably, the non-novel combination of SG and Talazoparib, a PARP inhibitor which is currently being tested with SG in clinical trials, was seen here as well (Fig. 5D, I) [65]. Based on these results and its translational potential, the SG + Everolimus combination was prioritized for further evaluation. Prior work from our group has demonstrated synergy between XPO1 inhibitors and mTOR blockade in TNBC models, including the carboplatin-insensitive PDX model WHIM2 [10]. For this reason combination of Selinexor (KPT-330), an XPO-1 inhibitor, and Everolimus were tested in parallel.

### In vivo validation of synergistic combinations in CR TNBC

**Fig. 6:**
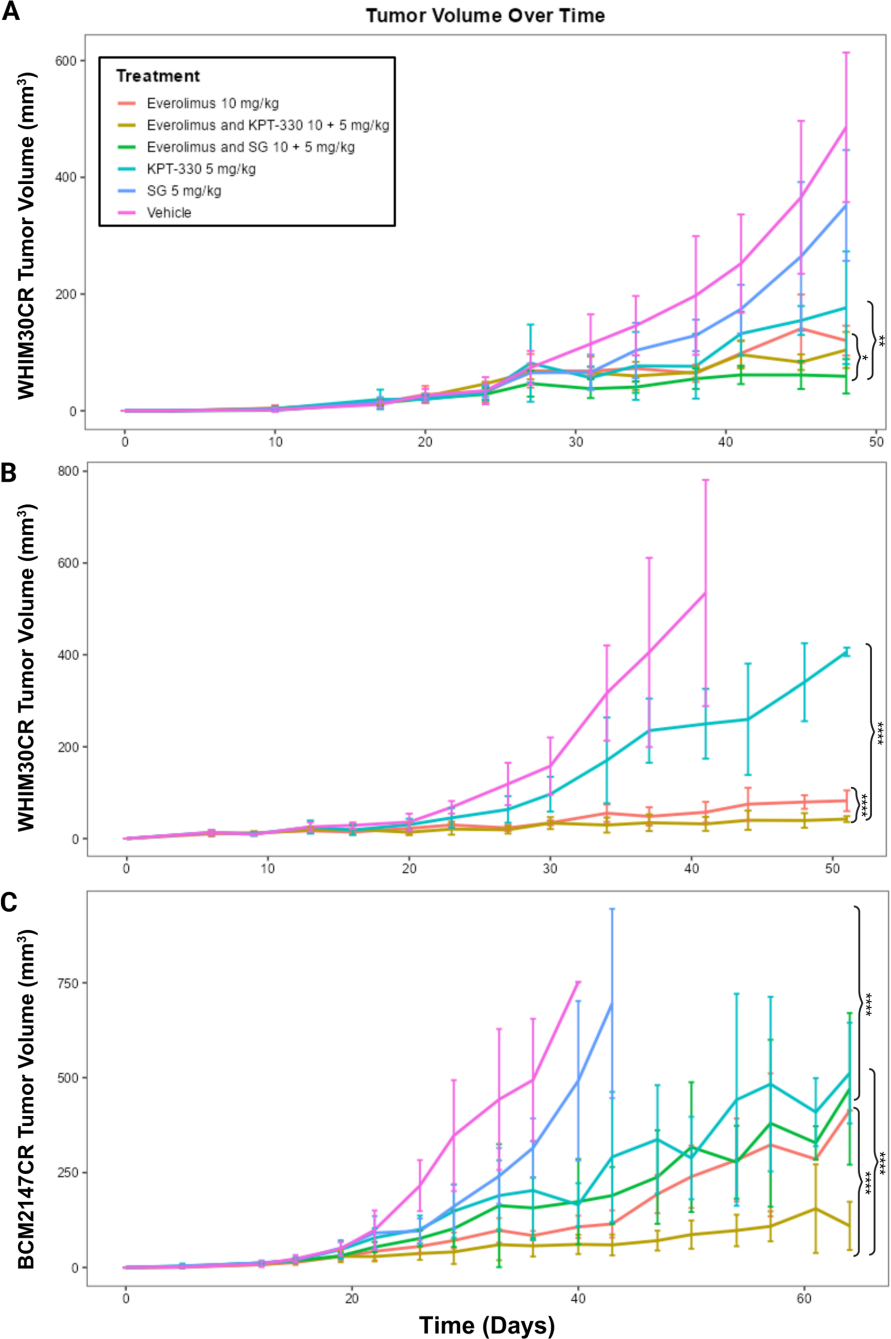
In vivo validation of SG- and Everolimus-based combinations in carboplatin-resistant PDX models. **A** Tumor growth curves from WHIM30CR mice treated with SG, Everolimus, KPT-330, or relavent combinations. **B** Expanded WHIM30CR cohort validating synergy of Everolimus + Selinexor (KPT-330). **C** BCM-2147CR model treated with SG + Everolimus or Everolimus + Selinexor or mono-agents, showing variable efficacy across models. Tumor volumes were measured twice weekly and analyzed by Welch’s two-sample t-test (A) or linear mixed-effects modeling (B-C). (**p < 0.01, ***p < 0.001, ****p < 0.0001)

Given the strong synergy observed in vitro, we next evaluated SG-based drug combinations in vivo using NSG mice bearing WHIM30CR and BCM2147CR tumors. In WHIM30CR SG + Everolimus showed significantly reduced tumor volume when compared to either drug alone in an initial cohort of three mice per group (Fig. 6A). Noticing a trend in the Everolimus + Selinexor (KPT-330) group, we sought to determine if cohort expansion would yield significant results. Expanding the cohort to 4 mice per group did result in a more robust findings with a significant difference noted in the combination group when compared to either mono-agent (Fig. 6B).

In BCM-2147CR we again tested Everolimus + Selinexor (KPT-330) and Everolimus + SG combinations (Fig. 6C). While Everolimus + Selinexor (KPT-330) showed a synergistic effect in this model, the Everolimus + SG combination did not show significant improvement over Everolimus alone, suggesting that this effect is being driven primarily by the Everolimus in this model. Across both models, Everolimus + SG consistently yielded the most robust effect size, supporting this combination as a leading candidate for overcoming carboplatin resistance in TNBC.

Additionally, we assessed blood taken from treatment mice without tumors and noted some shifts with treatment similar to those seen in patients, but did not note significant weight loss in the mice following treatment (Supplemental Fig. 4).

### Dual mTOR and XPO1 inhibition reduces primary and metastatic burden in WHIM2

**Fig. 7:**
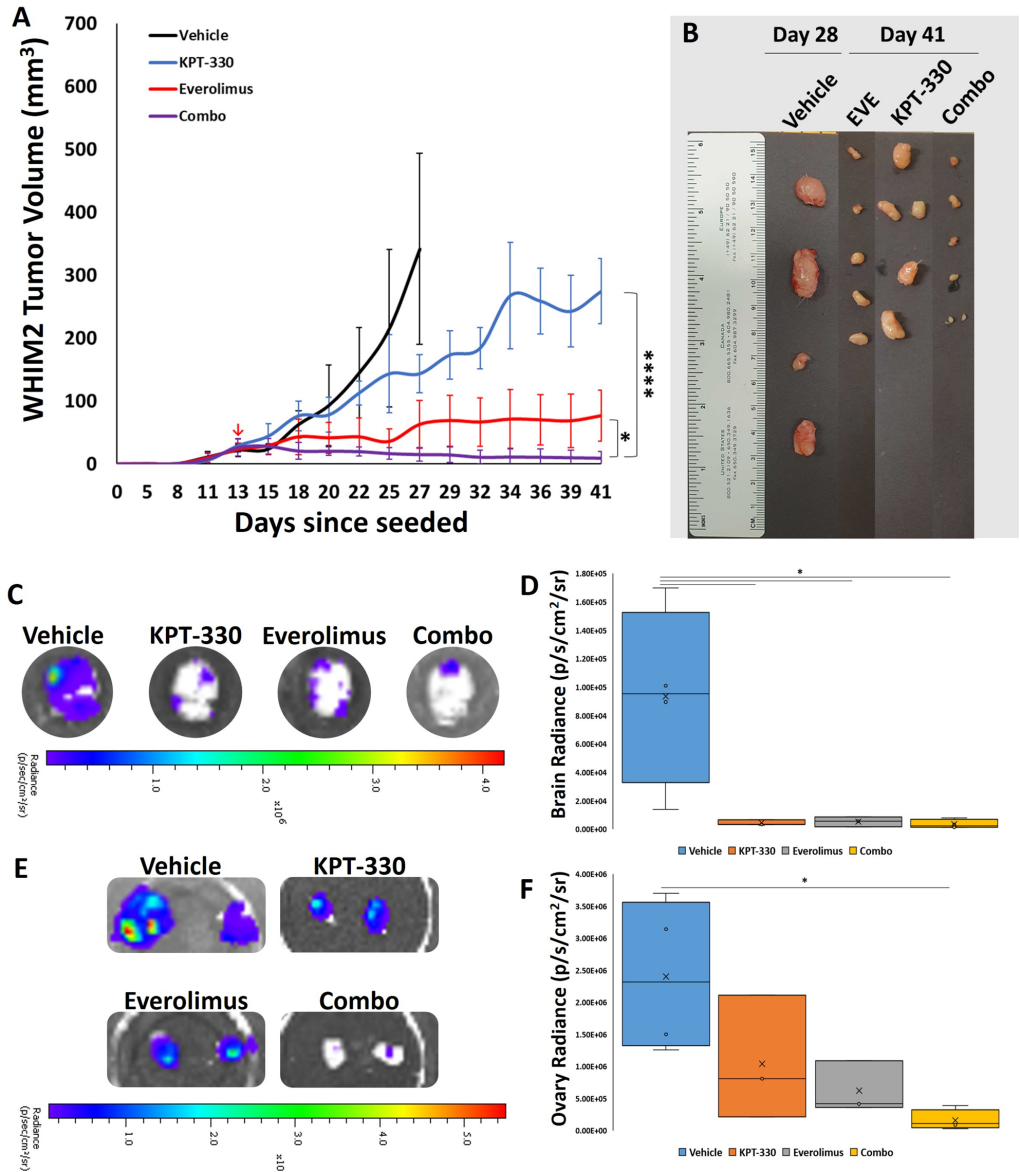
Mammary gland and metastasis studies of WHIM2 cancer burden over time after treatment with therapy. **A** In vivo tumor burden of WHIM2 mammary gland injected mice: after treatment with vehicle (black, *n* = 4), KPT-330 (blue, *n* = 4), everolimus (red, *n* = 5) or combo (purple, *n* = 5). Tumor burden was measured as tumor volume (y-axis, mm^3^) by days since seeded (x-axis). The red arrow indicates start of treatment administration. One-way ANOVA was performed to assess differences in tumor volume between combo and single-agent treatment groups, where *p* < 0.05 was considered statistically significant. **B** Excised WHIM2 tumor masses from mammary gland injected mice. **C-F** Ex vivo assessment of average WHIM2 metastasis burden at day 35 since seeding via intracardiac injection after four weeks of treatment with therapy. IVIS images of luciferin-cleaved radiance (p/sec/cm^2^/sr) in NSG mice burdened with WHIM2 (**C**) brain metastasis and (**E**) ovary metastasis at day 35 post-injection. Mice were treated either with vehicle (*n* = 4), KPT-330 (*n* = 3), everolimus (*n* = 3) or combo (*n* = 4). Red depicted the highest radiance and purple depicted the lowest radiance. Box and whisker plots of average radiances (p/sec/cm^2^/sr) of WHIM2 (**D**) brain metastasis burden and (**F**) ovary metastasis burden in vehicle (blue), KPT-330 (orange), everolimus (grey) and combo (yellow) mice. One-way ANOVA was performed to assess differences in total metastasis burden represented by average radiance between the vehicle group and the treatment groups, where *p* < 0.05 was considered statistically significant

To further evaluate the therapeutic potential of combined mTOR and XPO1 inhibition, WHIM2 PDX tumors were treated with Selinexor (KPT-330) and Everolimus. WHIM2 was selected due to its intrinsic carboplatin resistance and high XPO1 expression. In the mammary gland tumor (MGT) model, combination therapy resulted in a marked reduction in tumor volume compared with single-agent Selinexor, single-agent Everolimus, and vehicle controls (Fig. 7A–B). Tumors in the dual-treatment group demonstrated both delayed growth onset and sustained regression over the treatment course.

In mice bearing metastatic WHIM2 disease, the combination also significantly reduced metastatic burden. Quantitative imaging showed decreased whole-body signal relative to controls (Supplemental Fig. 5A; Supplemental Fig. 6). Organ-specific analyses confirmed reduced metastatic lesions in the brain (Fig. 7C–D; Supplemental Fig. 5B) and ovary (Fig. 7E–F; Supplemental Fig. 5C). Notably, in several mice, metastases in these organs were undetectable following dual treatment, whereas they remained prominent in vehicle and single-agent treated cohorts.

Together, these data demonstrate that combined inhibition of XPO1 and mTOR suppresses both primary tumor growth and metastatic dissemination in the WHIM2 model.

## Discussion

In this study, we established and comprehensively profiled isogenic CR TNBC PDX models to investigate mechanisms of resistance and identify potential therapeutic strategies. By generating matched CR/CS pairs from four genetically and phenotypically distinct TNBC PDX lines, we created a clinically relevant system to dissect tumor-intrinsic adaptations that emerge during carboplatin treatment. This model set represents a uniquely diverse and well-controlled resource for investigating platinum resistance in TNBC.

### Heterogeneity of resistance mechanisms across TNBC models

Our findings highlight the heterogeneity of molecular programs underlying carboplatin resistance. Single-cell and bulk transcriptomic analyses revealed distinct transcriptional changes in each CR/CS pair, with no single gene consistently altered across all models after multiple testing correction. Instead, subsets of resistance-associated genes were shared among two or more models, suggesting partial convergence of pathways rather than a universal mechanism. This aligns with prior observations that TNBC is a highly heterogeneous disease, both at baseline and under therapeutic pressure [23, 24, 66–68].

Despite this heterogeneity, several models demonstrated resistance programs linked to DNA repair capacity. In BCM-2147CR, we observed marked upregulation of BRCA1 at both RNA and protein levels, consistent with restoration of homologous recombination repair as a driver of acquired resistance [69, 70]. WHIM30 also demonstrated passage-dependent changes, with later CR passages showing increased BRCA1 expression, while some earlier passages displayed suppression of cancer-testis antigens HORMAD1 and MAGE-A4, both previously associated with HRD-high states [59, 71, 72]. In contrast, BCM-3887 and BCM-7482, which carry pathogenic BRCA1 mutations, exhibited distinct adaptive pathways. BCM-3887CR tumors upregulated mismatch repair genes (MLH1, MLH2, MLH3), suggesting compensation via alternative repair programs [59], while BCM-7482CR tumors showed pronounced induction of HORMAD1, consistent with increased tolerance of replication stress [72, 73]. Together, these results support the concept that platinum resistance in TNBC can arise through restoration of homologous recombination in BRCA1-wildtype tumors or through activation of alternative repair/tolerance pathways in BRCA1-deficient backgrounds.

### Proteomic profiling identifies markers but limited direct targets

Proteomic analysis of CR versus CS tumors in BCM-2147 and WHIM30 revealed several proteins differentially expressed in resistant tumors, including UCHL1. Although UCHL1 has been implicated as an oncogenic driver in breast cancer [62], functional validation in our models did not demonstrate therapeutic vulnerability worthy of further investigation. This suggests that while such proteins may serve as markers of resistance, they may not represent effective direct targets in this setting. These findings underscore the need for multi-level validation and highlight the importance of distinguishing between biomarkers of resistance and actionable dependencies.

### Therapeutic implications: exploiting vulnerabilities with combination strategies

Beyond mechanistic insights, these isogenic CR PDX models provide a platform for preclinical evaluation of rational therapeutic strategies. High-throughput drug screening identified consistent synergy between the standard-of-care ADC SG and inhibitors of the mTOR pathway. Notably, the non-novel combination of SG and PARP inhibitor also emerged as synergistic, supporting previous findings [65]. SG + Everolimus demonstrated robust synergy in vitro across multiple CR models and was further validated in vivo, where the combination significantly reduced tumor growth in WHIM30CR and BCM-2147CR models. While efficacy varied between models, the reproducibility of synergy with mTOR inhibition suggests this pathway may represent a tractable vulnerability in carboplatin-resistant TNBC.

We also evaluated the combination of Selinexor (KPT-330) and Everolimus. In WHIM2, an intrinsically carboplatin-resistant model with high XPO1 expression, dual inhibition markedly suppressed both primary tumor growth and metastatic dissemination. This is particularly notable given the limited number of therapeutic strategies available for metastatic TNBC and highlights the potential of XPO1/mTOR co-targeting to address both local and disseminated disease. This finding is supported in part by other studies which have noted synergistic effects of XPO1 inhibition with mTOR pathway [74].

### Limitations and future directions

Several limitations should be acknowledged. First, while the use of multiple genetically distinct TNBC PDX lines increases generalizability, resistance mechanisms observed here may still not capture the full spectrum present in patients [23, 24]. Second, although our omics profiling identified model-specific pathways, functional validation was limited for many candidates, and additional mechanistic studies will be required to establish causality. Third, in vivo combination studies were conducted with modest cohort sizes, which, while sufficient to identify significant effects, may underestimate variability. Finally, while Everolimus and Selinexor are clinically available, their tolerability in combination with ADCs such as SG in patients remains to be established.

## Conclusions

Together, these studies establish a robust platform of isogenic carboplatin-resistant TNBC PDX models and demonstrate their utility for dissecting mechanisms of resistance and testing rational drug combinations. Our findings emphasize the heterogeneity of resistance programs, reveal both BRCA1-dependent and -independent adaptations, and identify SG + mTOR inhibition as a promising therapeutic avenue. Moreover, dual mTOR and XPO1 inhibition demonstrates potent activity against both primary and metastatic disease in intrinsically resistant TNBC. These results provide both mechanistic insights and translational strategies to inform therapeutic development for patients with platinum-refractory TNBC.

## Supplementary Information

Supplementary Material 1. Figure S1: Nuclear area comparison between CS and CR pairs in BCM-7482. Significance determined by unpaired t-test (p-value < 0.0001). Figure was generated using Prism 10 by GraphPad.

Supplementary Material 2. Figure S2: BRCA1 immunohistochemistry staining with quantification. Representative images of BCM-2147 (A) and BCM-2147CR (B) IHC stains. (C) Quantification of differences between BCM-2147 and BCM-2147CR using linear mixed effect modelling (p-value ~ 0.08). Similarly images of BCM-3887 (D) and BCM-3887CR (E) IHC stains and (F) quantification of differences. BCM-7482 (G) and BCM-7482CR (H) IHC stains and (I) quantification of differences. WHIM30 (J) and WHIM30CR (K) IHC stains and (L) quantification of differences.

Supplementary Material 3. Figure S3: Immunohistochemistry quantification of differentially represented proteins in BCM-2147 & WHIM30 CS/CR pairs.

Supplementary Material 4. Figure S4: HEMAVET data results, blood analysis. Results for (A) percentage of neutrophils, (B) percentage of lymphocytes, (C) percentage of monocytes, (D) percentage of eosinophils, (E) percent of basophils, (F) total red blood cell count, (G) hemaglobin concentration, (H) hematacrit, (I) mean corpuscular volume, (J) mean corpuscular hemoglobin, (K) mean corpuscular hemoglobin concentration, and (L) total platelet count.

Supplementary Material 5. Figure S5: All in vivo and ex vivo images of average WHIM2 metastasis burden at day 35 since seeding after four weeks of treatment with therapy. IVIS images of luciferin-cleaved radiance (p/sec/cm2/sr) in NSG mice burdened with WHIM2 (A) total metastasis, (B) brain metastasis, and (C) ovary metastasis at day 35 since intracardiac injection. Mice were treated either with vehicle (n = 4), KPT-330 (n = 3), Everolimus (n = 3) or both drugs in combination, i.e. combo (n = 4). A radiance scalebar next to the images depicts radiance intensity, with red depicting the highest radiance and purple depicting the lowest radiance.

Supplementary Material 6. Figure S6: Total metastasis spread over time. IVIS images of luciferin-cleaved radiance (p/sec/cm^2^/sr) in NSG mice burdened with WHIM2 metastasis from day 0 through day 35. Mice were treated either with vehicle (*n* = 4), KPT-330 (*n* = 3), Everolimus (*n* = 3) or Combo (*n* = 4). A radiance scalebar next to the images depicts radiance intensity, with red depicting the highest radiance and purple depicting the lowest radiance.

Supplementary Material 7. Supplemental File 1: Contains data used in the generation of Figure S1 and masking scheme.

Supplementary Material 8. Supplemental File 2: Pathway enrichment results (Enrich and GSEA) for differentially expressed proteins in the WHIM30 carboplatin-resistant versus carboplatin-sensitive comparison. Worksheets are organized by analysis type and gene-set collection (e.g., Enrich.KEGG, GSEA.C7).

Supplementary Material 9. Supplemental File 3: Pathway enrichment results (Enrich and GSEA) for differentially expressed proteins in the BCM-2147 carboplatin-resistant versus carboplatin-sensitive comparison. Worksheets are organized by analysis type and gene-set collection (e.g., Enrich.KEGG, GSEA.C5).

Supplementary Material 10. Supplemental File 4: Differential gene expression analysis performed on proteomics data from the carboplatin-resistant versus carboplatin-sensitive comparison of BCM-2147 and WHIM30 models.

Supplementary Material 11. Supplemental File 5: Contains data used in the generation of Fig. 5A. Reported are coeffiecient of drug interaction (CDI) scores for screens involving treatment with these agents at 1 μm and SG at the ~ IC20 dose for that model (~ 2nM).

## Data Availability

Bulk and single cell RNA sequencing data associated with this publication can be found in the Gene Expression Omnibus (GEO) database under accession numbers GSE276609, GSE235169, GSE309616, and GSE309617.

## Abbreviations

ADC: Antibody–drug conjugate
ANOVA: Analysis of variance
ATCC: American Type Culture Collection
ClinVar: Clinical Variation database
CR: Carboplatin resistant
CS: Carboplatin sensitive
DEG: Differentially expressed gene
ER: Estrogen receptor
FFPE: Formalin-fixed paraffin-embedded
GEO: Gene Expression Omnibus
H&E: Hematoxylin and eosin
HRD: Homologous recombination deficiency
IHC: Immunohistochemistry
IVIS: In Vivo Imaging System
NSG: NOD scid gamma (mice)
PDX: Patient-derived xenograft
PR: Progesterone receptor
scRNA-seq: Single-cell RNA sequencing
SG: Sacituzumab govitecan
TNBC: Triple-negative breast cancer
TMT-MS: Tandem mass tag mass spectrometry
t-SNE: T-distributed stochastic neighbor embedding
UMAP: Uniform Manifold Approximation and Projection
UMI: Unique molecular identifier
VCF: Variant Call Format
