# Supplementary figures and images for: Therapeutic synergies that overcome carboplatin resistance in triple-negative breast cancer
Source: J Exp Clin Cancer Res. 2026 Feb 3;45:83. doi: 10.1186/s13046-025-03636-9 (PMC13037280; doi:10.1186/s13046-025-03636-9)

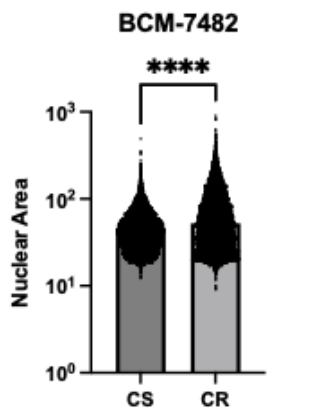

Supplement: Supplementary file 1 — Supplementary Material 1. Figure S1: Nuclear area comparison between CS and CR pairs in BCM-7482. Significance determined by unpaired t-test (p-value < 0.0001). Figure was generated using Prism 10 by GraphPad. [file 13046_2025_3636_MOESM1_ESM.png]

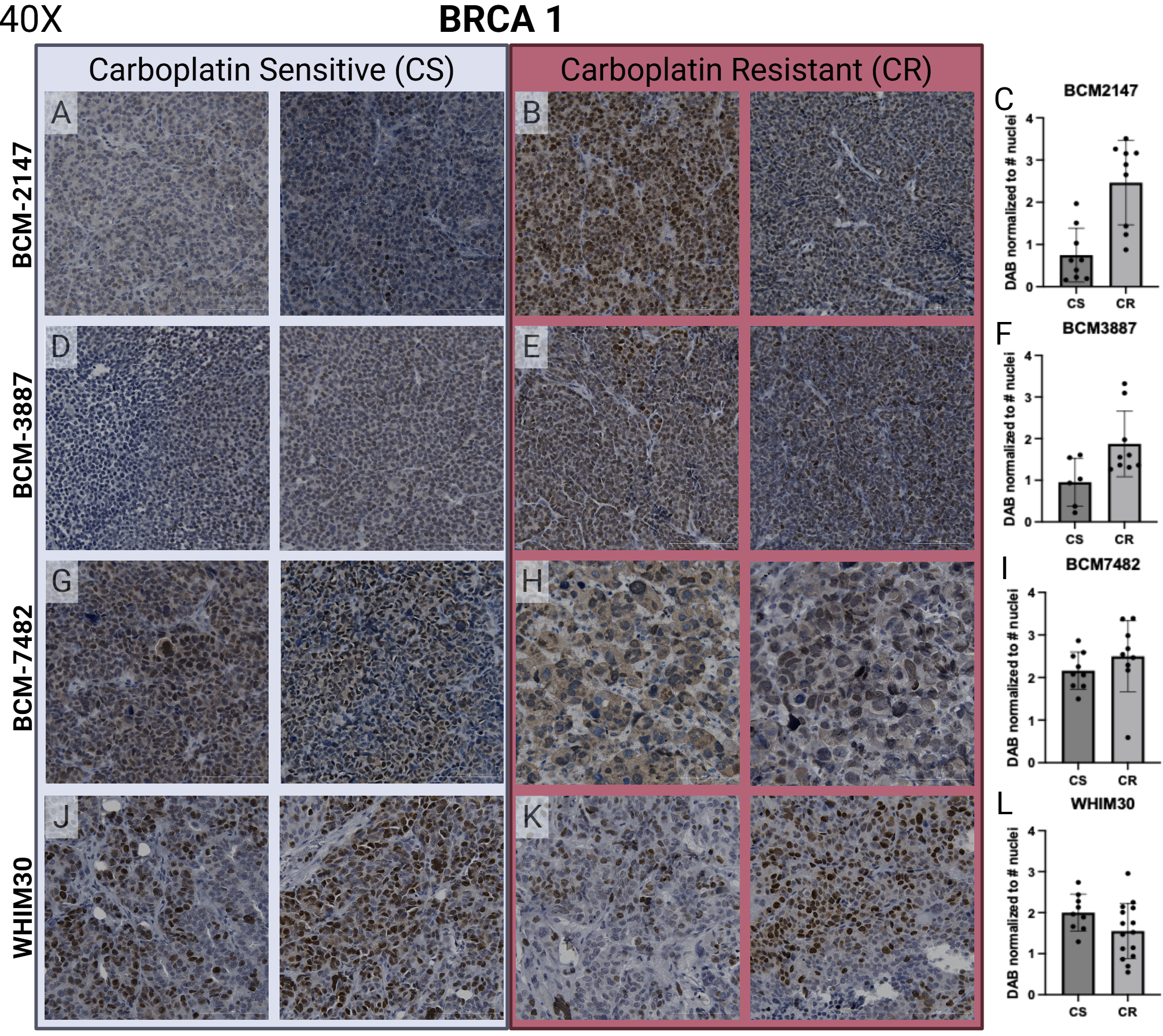

Supplement: Supplementary file 2 — Supplementary Material 2. Figure S2: BRCA1 immunohistochemistry staining with quantification. Representative images of BCM-2147 (A) and BCM-2147CR (B) IHC stains. (C) Quantification of differences between BCM-2147 and BCM-2147CR using linear mixed effect modelling (p-value ~ 0.08). Similarly images of BCM-3887 (D) and BCM-3887CR (E) IHC stains and (F) quantification of differences. BCM-7482 (G) and BCM-7482CR (H) IHC stains and (I) quantification of differences. WHIM30 (J) and WHIM30CR (K) IHC stains and (L) quantification of differences. [file 13046_2025_3636_MOESM2_ESM.png]

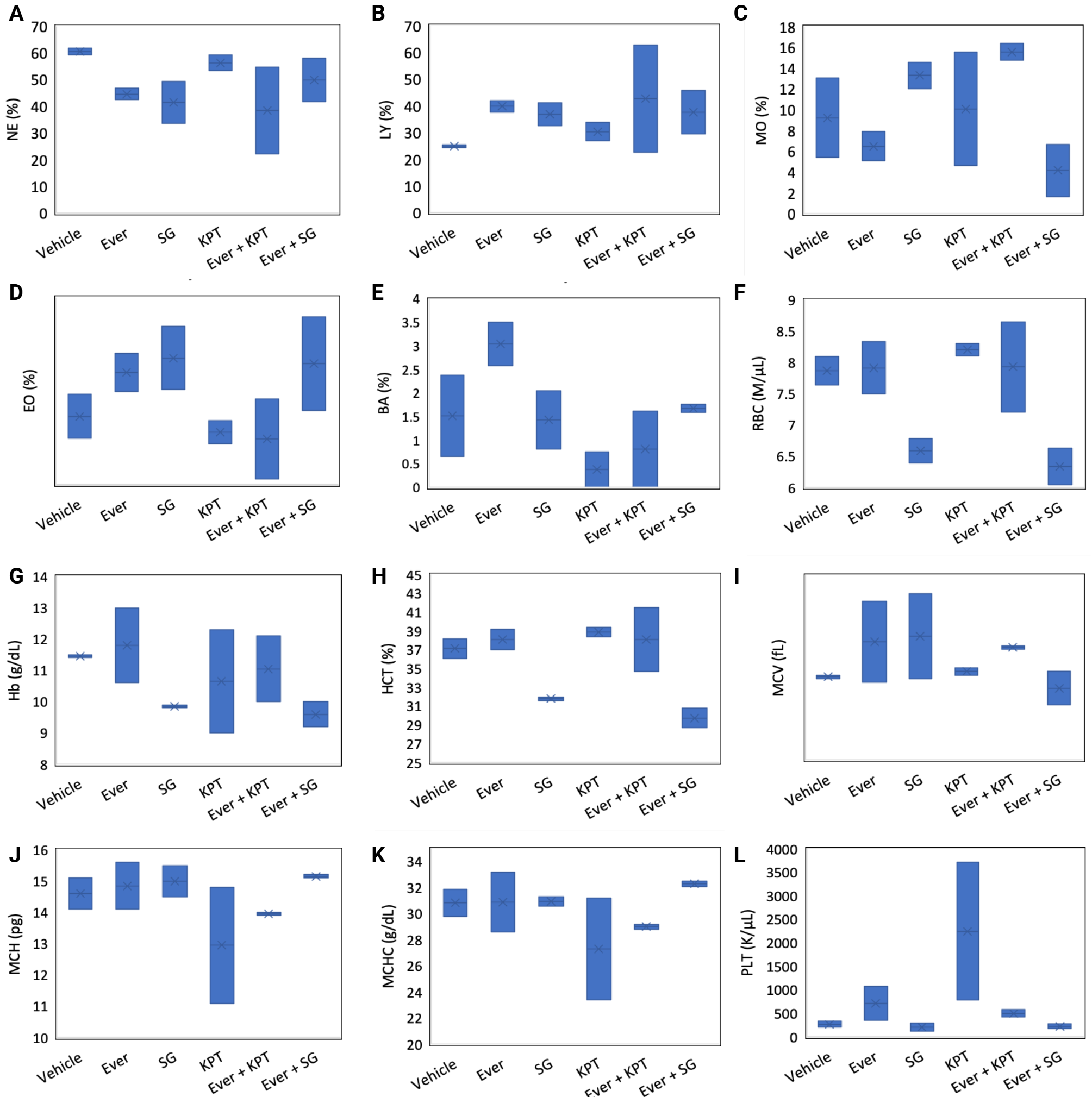

Supplement: Supplementary file 4 — Supplementary Material 4. Figure S4: HEMAVET data results, blood analysis. Results for (A) percentage of neutrophils, (B) percentage of lymphocytes, (C) percentage of monocytes, (D) percentage of eosinophils, (E) percent of basophils, (F) total red blood cell count, (G) hemaglobin concentration, (H) hematacrit, (I) mean corpuscular volume, (J) mean corpuscular hemoglobin, (K) mean corpuscular hemoglobin concentration, and (L) total platelet count. [file 13046_2025_3636_MOESM4_ESM.png]

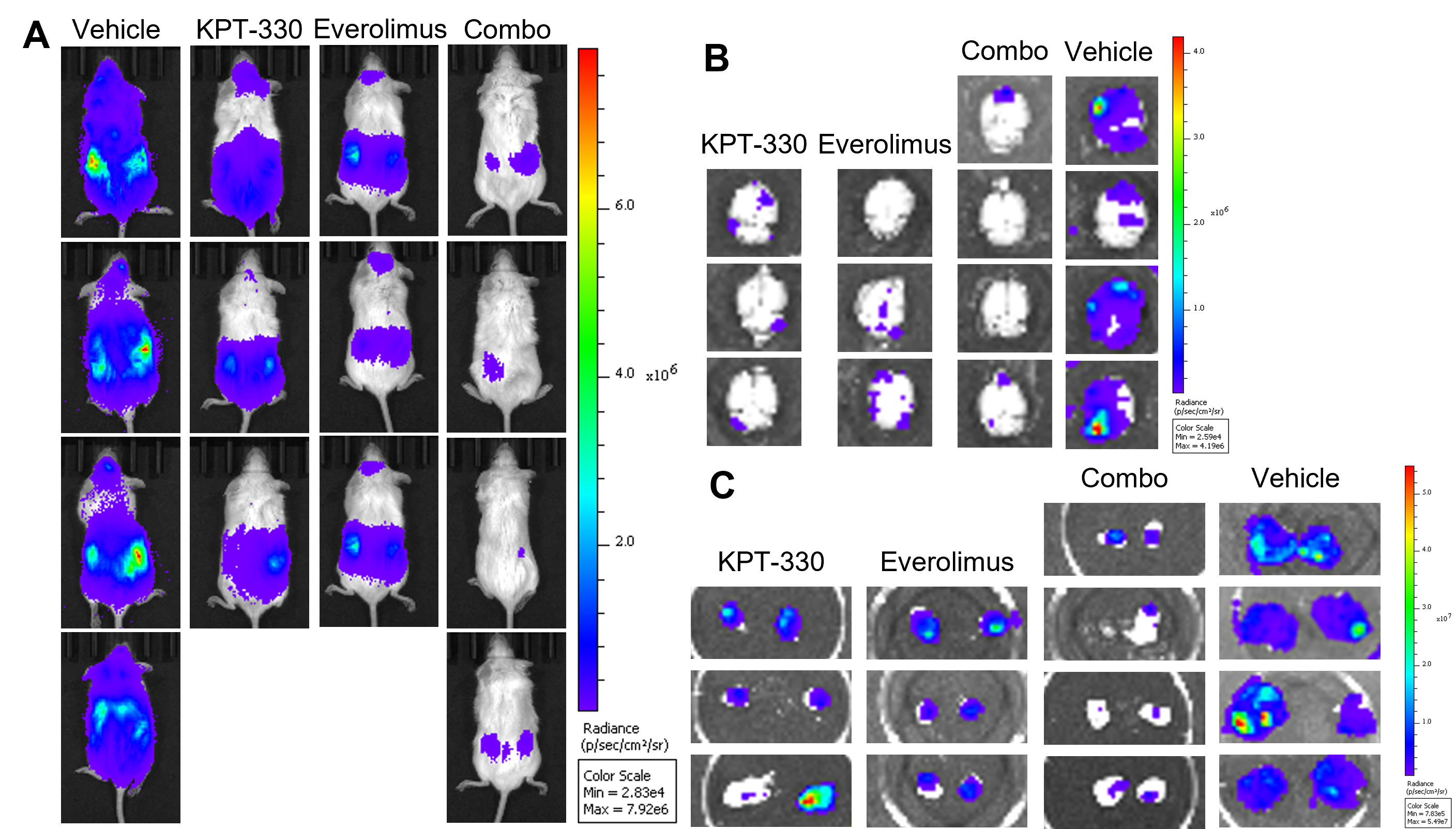

Supplement: Supplementary file 5 — Supplementary Material 5. Figure S5: All in vivo and ex vivo images of average WHIM2 metastasis burden at day 35 since seeding after four weeks of treatment with therapy. IVIS images of luciferin-cleaved radiance (p/sec/cm2/sr) in NSG mice burdened with WHIM2 (A) total metastasis, (B) brain metastasis, and (C) ovary metastasis at day 35 since intracardiac injection. Mice were treated either with vehicle (n = 4), KPT-330 (n = 3), Everolimus (n = 3) or both drugs in combination, i.e. combo (n = 4). A radiance scalebar next to the images depicts radiance intensity, with red depicting the highest radiance and purple depicting the lowest radiance. [file 13046_2025_3636_MOESM5_ESM.png]

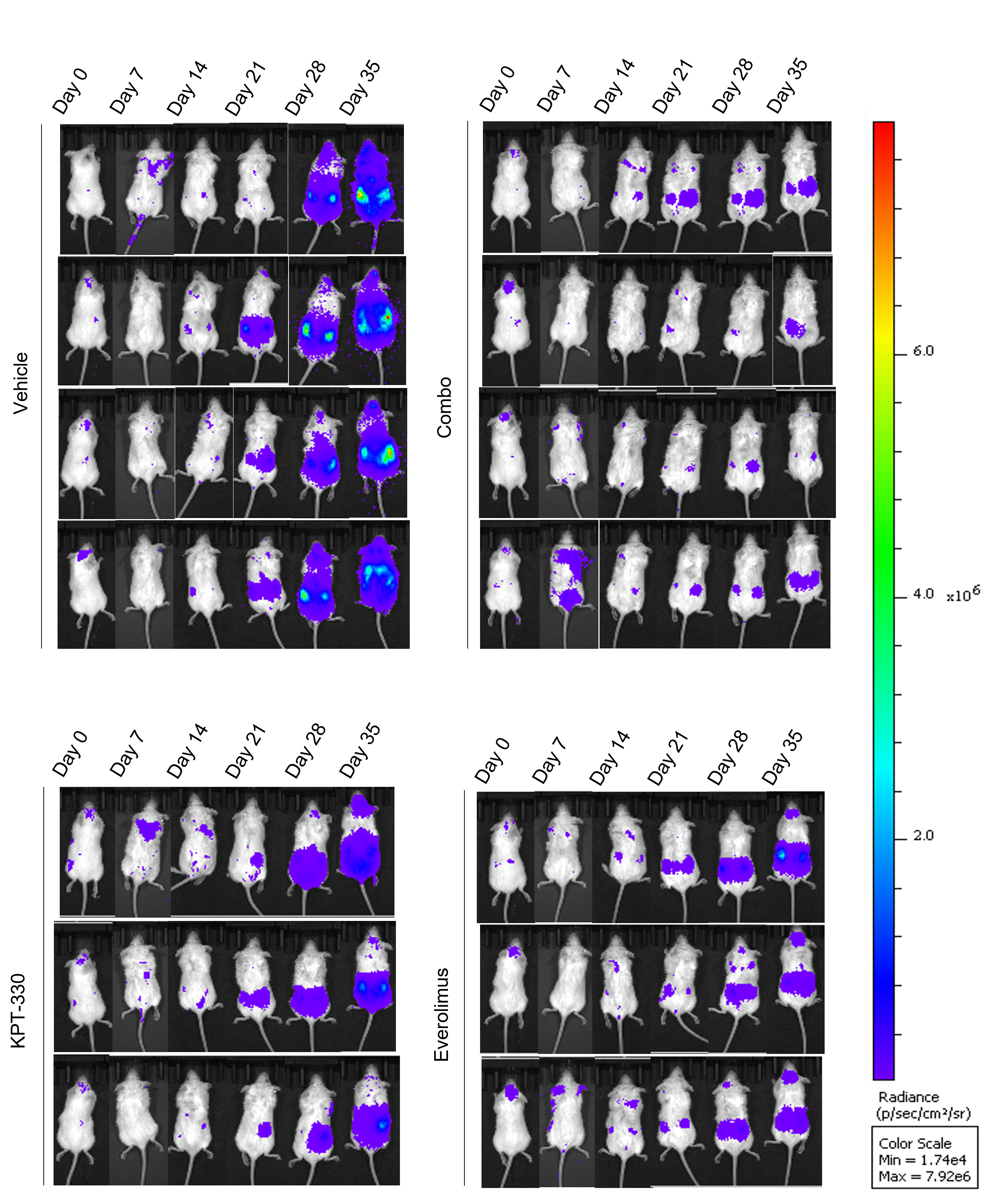

Supplement: Supplementary file 6 — Supplementary Material 6. Figure S6: Total metastasis spread over time. IVIS images of luciferin-cleaved radiance (p/sec/cm2/sr) in NSG mice burdened with WHIM2 metastasis from day 0 through day 35. Mice were treated either with vehicle (n = 4), KPT-330 (n = 3), Everolimus (n = 3) or Combo (n = 4). A radiance scalebar next to the images depicts radiance intensity, with red depicting the highest radiance and purple depicting the lowest radiance. [file 13046_2025_3636_MOESM6_ESM.png]
