# Supplementary material for: Therapeutic synergies that overcome carboplatin resistance in triple-negative breast cancer
Source: J Exp Clin Cancer Res. 2026 Feb 3;45:83. doi: 10.1186/s13046-025-03636-9 (PMC13037280; doi:10.1186/s13046-025-03636-9)
Supplement: Supplementary file 3 — Supplementary Material 3. Figure S3: Immunohistochemistry quantification of differentially represented proteins in BCM-2147 & WHIM30 CS/CR pairs. [file 13046_2025_3636_MOESM3_ESM.pdf]

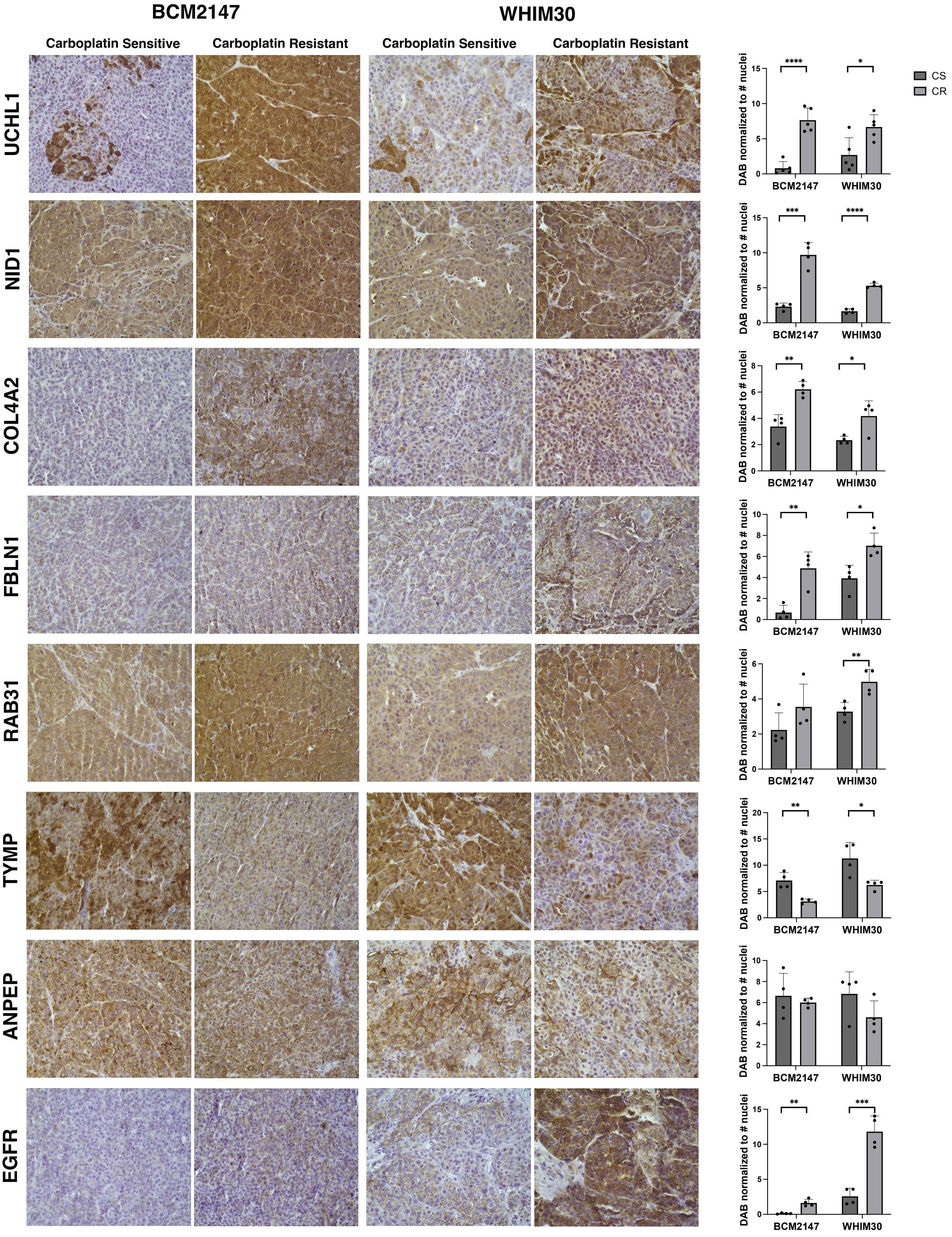

BCM2147
WHIM30
Carboplatin Sensitive
Carboplatin Resistant
Carboplatin Sensitive
Carboplatin Resistant
UCHL1
NID1
COL4A2
FBLN1
RAB31
TYMP
ANPEP
EGFR
DAB normalized to # nuclei
BCM2147
WHIM30
CS
CR
****
*
***
****
**
*
**
*
**
**
*
**
***
